# Long non-coding RNA HOTAIR promotes exosome secretion by regulating RAB35 and SNAP23 in hepatocellular carcinoma

**DOI:** 10.1186/s12943-019-0990-6

**Published:** 2019-04-03

**Authors:** Liang Yang, Xueqiang Peng, Yan Li, Xiaodong Zhang, Yingbo Ma, Chunli Wu, Qing Fan, Shibo Wei, Hangyu Li, Jingang Liu

**Affiliations:** Department of General Surgery, The Fourth Affiliated Hospital, China Medical University, Shenyang, 110032 China

**Keywords:** HOTAIR, Exosome, RAB35, SNAP23, HCC

## Abstract

**Background:**

Emerging evidence indicates that tumor cells release a large amount of exosomes loaded with cargos during tumorigenesis. Exosome secretion is a multi-step process regulated by certain related molecules. Long non-coding RNAs (lncRNAs) play an important role in hepatocellular carcinoma (HCC) progression. However, the role of lncRNA HOTAIR in regulating exosome secretion in HCC cells remains unclear.

**Methods:**

We analyzed the relationship between HOTAIR expression and exosome secretion-related genes using gene set enrichment analysis (GSEA). Nanoparticle tracking analysis was performed to validate the effect of HOTAIR on exosome secretion. The transport of multivesicular bodies (MVBs) after overexpression of HOTAIR was detected by transmission electron microscopy and confocal microscopy analysis of cluster determinant 63 (CD63) with synaptosome associated protein 23 (SNAP23). The mechanism of HOTAIR’s regulation of Ras-related protein Rab-35 (RAB35), vesicle associated membrane protein 3 (VAMP3), and SNAP23 was assessed using confocal co-localization analysis, phosphorylation assays, and rescue experiments.

**Results:**

We found an enrichment of exosome secretion-related genes in the HOTAIR high expression group. HOTAIR promoted the release of exosomes by inducing MVB transport to the plasma membrane. HOTAIR regulated RAB35 expression and localization, which controlled the docking process. Moreover, HOTAIR facilitated the final step of fusion by influencing VAMP3 and SNAP23 colocalization. In addition, we validated that HOTAIR induced the phosphorylation of SNAP23 via mammalian target of rapamycin (mTOR) signaling.

**Conclusion:**

Our study demonstrated a novel function of lncRNA HOTAIR in promoting exosome secretion from HCC cells and provided a new understanding of lncRNAs in tumor cell biology.

**Electronic supplementary material:**

The online version of this article (10.1186/s12943-019-0990-6) contains supplementary material, which is available to authorized users.

## Background

Hepatocellular carcinoma (HCC) is one of the most common cancers, with limited therapeutic options and poor prognosis [1]. Tumor development is not only determined by cancer cells, but also is regulated by the microenvironment [2]. The tumor microenvironment has complex matrix components and plays a vital role in tumor progression, such as growth, metastasis, and occurrence [3]. As the main category of extracellular vesicles, exosomes are double-layered vesicles of 30–150 nm in diameter, containing nucleic acids, proteins, and lipids to mediate intercellular communication [4]. A growing number of studies have indicated that exosomes can influence HCC progression from multiple aspects, such as angiogenesis, chemoresistance, metastasis, and the immune response [5]. In addition, measurement of exosome contents as biomarkers in HCC was revealed to be useful for early diagnosis and progression monitoring [6]. These studies have shed light on the potential functions of exosomes in the tumor microenvironment and accumulating evidence has shown that tumor cells release large amounts of exosomes loaded with cargos during tumorigenesis [7, 8]; however, the molecular mechanisms of exosome secretion in tumor cells remain unclear.

The biogenesis of exosomes is generated from the inward budding of the membranes of multivesicular bodies (MVBs) to form intraluminal vesicles (ILVs), which finally mature and are contained within MVBs [9]. This process involves various sorting machineries, including endosomal sorting complex required for transport (ESCRT)-dependent and ESCRT-independent processes [10]. The MVBs containing ILVs are transported along microtubules and fuse with the plasma membrane, causing the ILVs to be released outside the cell as exosomes. Before exosome release, multiple intracellular trafficking steps are required to regulate MVB motility, docking, and fusion with the plasma membrane, which involve different effectors and molecular mechanisms [11]. Rab GTPases are required for the motility of MVBs and docking at the plasma membrane [12]. Rab GTPases control MVBs transport by acting as molecular switches that convert between the GTP-bound active form and the GDP-bound inactive form [13]. The Rab family comprises almost 70 subtypes that show varied subcellular distributions. Several Rab GTPases play different roles in the exosomal pathway and mediate exosome secretion [14, 15]. Different Rab GTPases localize in distinct subcellular locations and are specific to cell types; however, the molecular mechanisms of Rab GTPases’ effects on exosome secretion in tumor cells require further study.

MVB fusion with the plasma membrane results in the release of ILVs as exosomes, which is the final and key step of exosome secretion. Soluble N-ethylmaleimide-sensitive fusion factor attachment protein receptor (SNARE) proteins have been shown to mediate this final step [16]. The SNARE complex involved in exocytic release comprises v-SNAREs on membranes of the MVBs and t-SNAREs on the cell membrane, forming a stable ternary complex that mediates exosome secretion. As v-SNAREs, vesicle-associated membrane protein (VAMP) 2, VAMP3, VAMP7, and VAMP8 are known to regulate exocytosis in tumor cells [17, 18]. In addition, synaptosome associated protein 23 (SNAP) 23 is an important t-SNARE that is mainly located at the plasma membrane [19]. Phosphorylation of SNAP23 not only promotes the formation of the SNARE complex, but also increases exosome secretion [20]. However, whether long non-coding RNAs (lncRNAs) participate in the regulation of SNARE proteins and mediate exosome secretion is unclear. HOX transcript antisense RNA (HOTAIR) is a 2158 nucleotide lncRNA transcribed from the HOXC locus and is frequently upregulated in many types of human cancer, including HCC [21]. Previously, we demonstrated that HOTAIR could induce autophagy by upregulating autophagy related 3 (ATG3) and ATG7 expression in HCC cells [22]. Although a growing number of studies revealed that HOTAIR could influence multiple biological functions as an oncogene [23], a relationship between lncRNA HOTAIR and exosome secretion has not been identified. To determine whether HOTAIR contributes to exosome secretion in HCC cells, we analyzed the function of HOTAIR in exosome secretion using nanoparticle tracking analysis (NTA). We demonstrated that HOTAIR could promote the release of exosomes from HCC cells. Given the importance of Rab GTPases and SNAREs in the mediation of exosome secretion, our mechanistic study revealed that HOTAIR facilitates the transport of MVBs towards the plasma membrane by regulating Ras-related protein Rab-35 (RAB35), which controls the docking process. Furthermore, we identified that HOTAIR promotes the colocalization of VAMP3 with SNAP23, which influences SNARE complex formation, leading to MVB fusion with the plasma membrane. Our research demonstrated the role of HOTAIR in exosome secretion and provides a new understanding of lncRNAs in tumor cell biology.

## Methods

### Gene set enrichment analysis (GSEA)

Liver hepatocellular carcinoma (LIHC) RNA sequencing (RNA-seq) data (374 cases) and normal tissues RNA-seq data (50 cases) were generated from The Cancer Genome Atlas (TCGA). The 374 cases were categorized into a HOTAIR high expression group and a HOTAIR low expression group. We performed GSEA analysis using GSEA v2.0 software (http://software.broadinstitute.org/gsea/index.jsp). Statistical significance was assessed by comparing the enrichment score with the enrichment results generated from 1000 random permutations of the gene set to obtain *p*-values.

### Cell culture

HepG2 (an HCC cells line) was obtained from Zhong Qiao Xin Zhou (Shanghai, China). The cell line was maintained in minimal essential medium (MEM) (HyClone, Logan, UT, USA) with 10% fetal bovine serum (HyClone) containing 100 U/mL penicillin and 100 μg/mL streptomycin and incubated at 37 °C under 5% CO_2_. Cells or culture medium were collected for experimentation at the indicated times.

### Cell transfection

All the HOTAIR plasmids and small interfering RNAs (siRNAs) were purchased from Genechem (Shanghai, China). HepG2 cell transfection was performed in 6-well plates using Invitrogen Lipofectamine 3000 (Thermo Fisher Scientific, Shanghai, China) according to the manufacturer’s instructions. At 48 h after transfection, the cells or culture supernatants were harvested to perform further experiments. The full length cDNA sequences of HOTAIR were cloned into pcDNA3.1(GV144) vector (GeneChem Shanghai, China) to construct HOTAIR overexpression plasmid (pcDNA-HOTAIR). The two siRNA sequence for RAB35 was as follows: Rab35 siRNA#1: 5′-CTGGTCCTCCGAGCAAAGAAA-3′. Rab35 siRNA#2: 5′-GAUGAUGUGUGCCGAAUAU-3′.

### Exosome isolation

Exosomes were prepared from the culture supernatant from a 48-h culture of HepG2 cells. The culture supernatant was centrifuged (Beckman XPN-100) at 2000×g for 10 min (4 °C) and 1000×g for 30 min (4 °C) to remove debris. Then, the supernatant was centrifuged at 100,000×g for 70 min (4 °C). The pellet was resuspended and washed in phosphate-buffered saline (PBS) and the supernatant was centrifuged at 100,000×g for 70 min (4 °C) again [24]. Finally, the exosomes were collected from the pellet, washed, and resuspended in PBS as described previously [24].

### Nanoparticle tracking analysis

The number and size of the exosomes were directly tracked by the rate of Brownian motion of exosomes using the NanoSight NS 300 system (NanoSight Technology, Malvern, UK), configured with a high-sensitivity sCMOS camera, fast video capture, and particle-tracking software (NanoSight, Amesbury, UK). The samples were diluted 150–3000 times with Dulbecco’s PBS (DPBS) without any nanoparticles to attain a concentration of 1–20 × 10^8^ particles per milliliter for analysis. Each sample was measured in triplicate at the camera, which recorded and tracked each visible particle. Exosome numbers and size distribution were explored using the Stokes-Einstein equation.

### Phos-tag SDS-PAGE and western blotting

Cells and exosomes were lysed in Radioimmunoprecipitation assay (RIPA) buffer containing protease inhibitors stored at − 20 °C until use. The proteins were separated by SDS-PAGE and then transferred to polyvinylidene fluoride (PVDF membranes. Trichloroacetic acids (TCA) precipitation was used to reduce the impurity content in the Phos-tag SDS-PAGE (#193–16,711) samples. Metal ions (Mn2+ or Zn2+) were removed from the gel using ethylenediaminetetraacetic acid (EDTA) before film transfer. After incubation with the appropriate primary antibodies, horseradish peroxidase-conjugated secondary antibodies were incubated with the membranes. The Li-Cor Odyssey protein imaging system was used to analyze protein bands. Antibodies used were: rabbit polyclonal anti-tumor susceptibility 101 (TSG101) (#14497–1-AP), rabbit polyclonal anti-cluster determinant 63 (CD63) (#25682–1-AP), rabbit polyclonal anti-RAB35 (#11329–2-AP), rabbit polyclonal anti-β-Actin (#60008–1-Ig), rabbit polyclonal anti-SNAP23 (#ab4114), rabbit polyclonal anti-mammalian target of rapamycin (mTOR) (#ABP54398), and rabbit polyclonal anti-mTOR (phospho Ser2448) (#ABP50363).

### Real-time PCR

We used the TRIzol reagent (Invitrogen) to extract total RNA from the control and treated cells, as previously described [22]. The RNA was reversely transcribed to cDNA using a Reverse Transcription Kit (Takara, Dalian, China). Real-time PCR was conducted using the SYBR-Green PCR Master Mix kit. The following primers were used: RAB5 forward 5′-AGACCCAACGGGCCAAATAC-3′, and reverse, 5′-GCCCCAATGGTACTCTCTTGAA-3′; RAB7 forward 5′-CTCATTATCGTCGGAGCCATTG-3′, and reverse, 5′-AGTGTGGTCTGGTATTCCTCATA-3′; RAB11 forward 5′-GCTCGGCCTCGACAAGTTC-3′, and reverse, 5′-ACTTATACCACTGCGTCTTCCT-3′; RAB27A forward 5′-GGAGAGGTTTCGTAGCTTAACG-3′, and reverse, 5′-CCACACAGCACTATATCTGGGT-3′; RAB27B forward 5′-TAGACTTTCGGGAAAAACGTGTG-3′, and reverse, 5′-AGAAGCTCTGTTGACTGGTGA-3′; RAB35 forward 5′-TTAAGCTTCGATGGCCCGGGACTACGACC-3′, and reverse, 5′-TTGGATCCTTAGCAGCAGCGTTTCTTTCGTTTACTG-3′; glyceraldehyde-3-phosphate dehydrogenase (GAPDH) forward 5′-AAAGATGTGCTTCGAGATGTGT-3′, and reverse, 5′-CACTTTGTCAGTTACCAACGTCA-3′. GAPDH was used as an endogenous control.

### Immunofluorescence and confocal microscopy

For immunofluorescence assays, cells were fixed with 4% paraformaldehyde for 25 min at 25 °C and then stained with the indicated primary antibodies (1:100) at 4 °C overnight. Cells were then incubated with secondary antibodies for 1 h at 37 °C. Finally, nuclei were stained with 2-(4-amidinophenyl)-1H-indole-6-carboxamidine (DAPI) (Beyotime) for 3 min at room temperature. Immunofluorescence was captured using a Nikon A1r confocal microscope. The antibodies used were as follows: rabbit polyclonal anti-SNAP23 (#ab4114), rabbit polyclonal anti-RAB35 (#11329–2-AP), mouse polyclonal anti-CD63 (#GTX28219), mouse polyclonal anti-VAMP3 (#66488–1-Ig), Goat anti-rabbit IgG H&L (fluorescein isothiocyanate (FITC)) (#ab6717), and Goat anti-mouse IgG H&L (Cy3) (#ab97035).

### Transmission electron microscopy

Samples of cells were quickly removed and fixed in 5% glutaraldehyde (protected from light) in 0.1 M phosphate buffer at 4 °C. The fixed cells were then subjected ultracryomicrotomy to generate slices of about 70 nm in thickness. The isolated exosomes (20–40 μm) in heavy suspension droplets were placed on the special copper mesh of the electron microscope, and then subjected to negative staining with 20 μL 2% phosphotungstic acid for 10 min. All samples were analyzed using a H-7650 electron microscope at 100KV.

### RNA immunoprecipitation (RIP) assay

RIP assay was performed using RNA-Binding Protein Immunoprecipitation Kit (Millipore, Bedford, MA) according to the manufacturer’s instructions. Cell lysates were incubated with RIP buffer containing magnetic beads conjugated with RAB35 antibody or negative control IgG (Millipore). Immunoprecipitated RNA was puried, and then subjected to real-time PCR analysis to detect the relative levels of HOTAIR.

### Pull-down assay

Pull-down assay was was examined using Pierce Magnetic RNA-Protein Pull-Down Kit (Thermo fisher) according to the manufacturer’s protocols. Biotin-labeled HOTAIR or antisense RNA was co-incubated with protein extract of HepG2 cells and magnetic bead. The retrieved protein was detected by western blot with β-actin as control.

### Statistical analysis

All data are presented as the mean ± standard error (SD) from three independent experiments, analyzed by SPSS version 17.0 software (IBM Corp., Armonk, NY, USA). Student’s t-test or analysis of variance were used to perform the statistical analyses. Statistically significance was concluded at *P < 0.05, **P < 0.01.

## Results

### Abnormal expression of HOTAIR is associated with exosome secretion

**Fig. 1:**
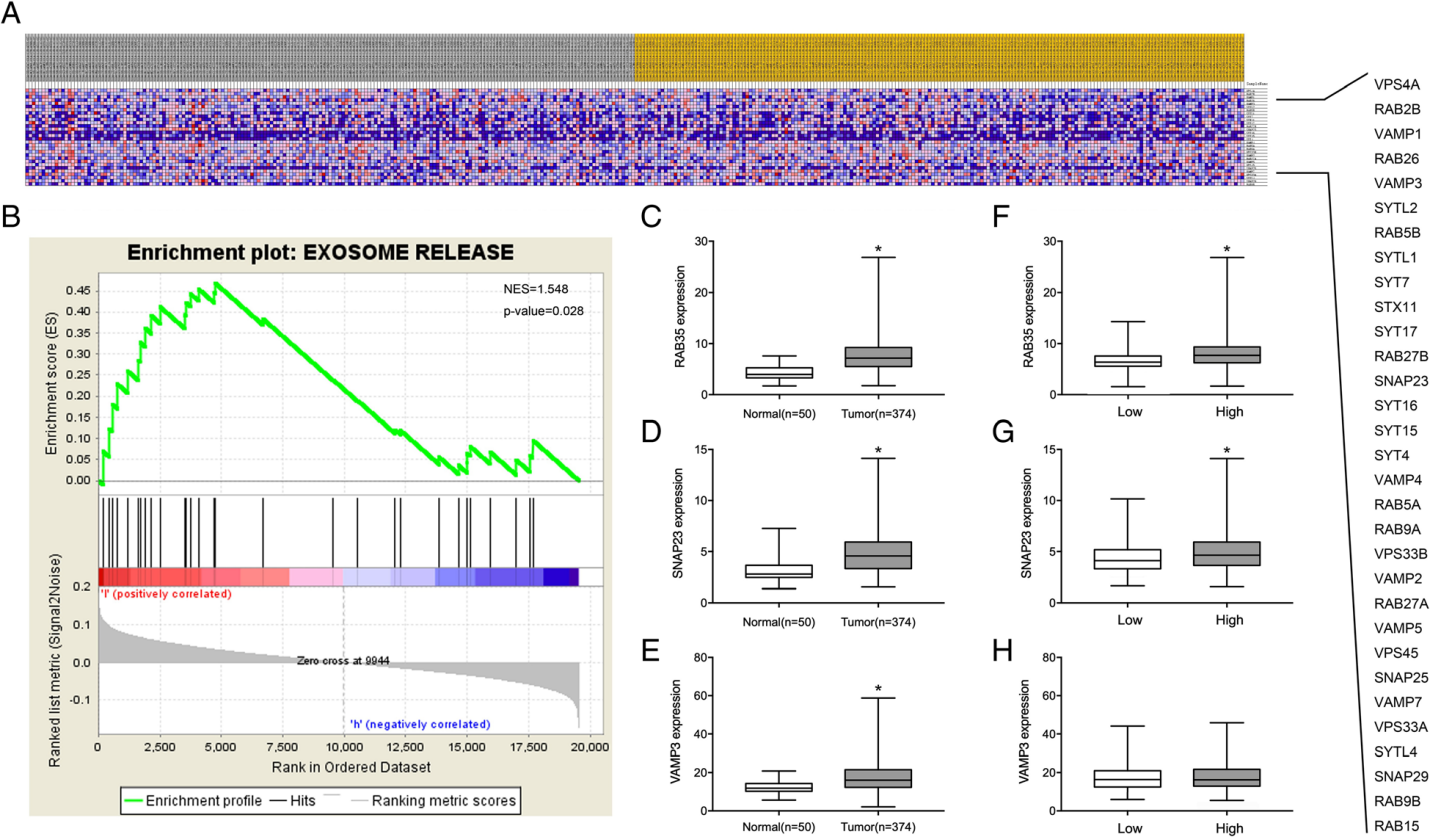
Abnormal expression of HOTAIR is associated with exosome secretion. **a** Heatmap showing the relative expression values for 31 exosome secretion-related genes in a series of 374 cases of liver cancer generated from TCGA and categorized into subgroups according to their median HOTAIR expression. The list on the right shows 31 genes involved in exosome secretion. **b** Enrichment plot showing enrichment of exosome secretion-related genes in the HOTAIR high expression group. **c**-**e** The mRNA expression of RAB35, SNAP23, and VAMP3 were analyzed in HCC tissues compared with normal tissues. **f**-**h** The mRNA expression of RAB35, SNAP23, and VAMP3 were analyzed in HOTAIR relative high group compared with HOTAIR relative low group.t-test *P-value < 0.05

Recent evidence shows that exosome secretion is a multi-step process regulated by certain related molecules [11]. Our previous review and other studies have summarized a series of regulators involved in exosome secretion [5, 11]. However, very little is known about the relationship between lncRNA HOTAIR and exosome secretion. We aimed to study the link between HOTAIR and exosome secretion-related genes. To date, 31 genes encoding proteins that regulate the process of exosome secretion have been identified, which are listed in Fig. 1a. We used the RNA-seq data from 374 cases of liver cancer generated from TCGA and categorized these cases into two groups: The low HOTAIR expression group (less than the median) and the high HOTAIR expression group (greater than the median). Using GSEA, we identified a significant enrichment of exosome secretion-related genes in the patients with HCC in the high HOTAIR group (normalized enrichment score (NES) = 1.548, *p*-value = 0.028, Fig. 1b). In addition, we showed that the expression levels of RAB35, SNAP23, and VAMP3 were significantly higher in HCC tissues than in normal tissues (Fig. 1c–e). Moreover, we divided the HCC tissues into relative high HOTAIR group and relative low HOTAIR group. We found that he expression levels of RAB35, SNAP23 were increased in HOTAIR high group (Fig. 1f–h). These results suggested that HOTAIR might play a role in regulating exosome secretion in HCC.

### HOTAIR promotes exosome secretion in HCC cells

**Fig. 2:**
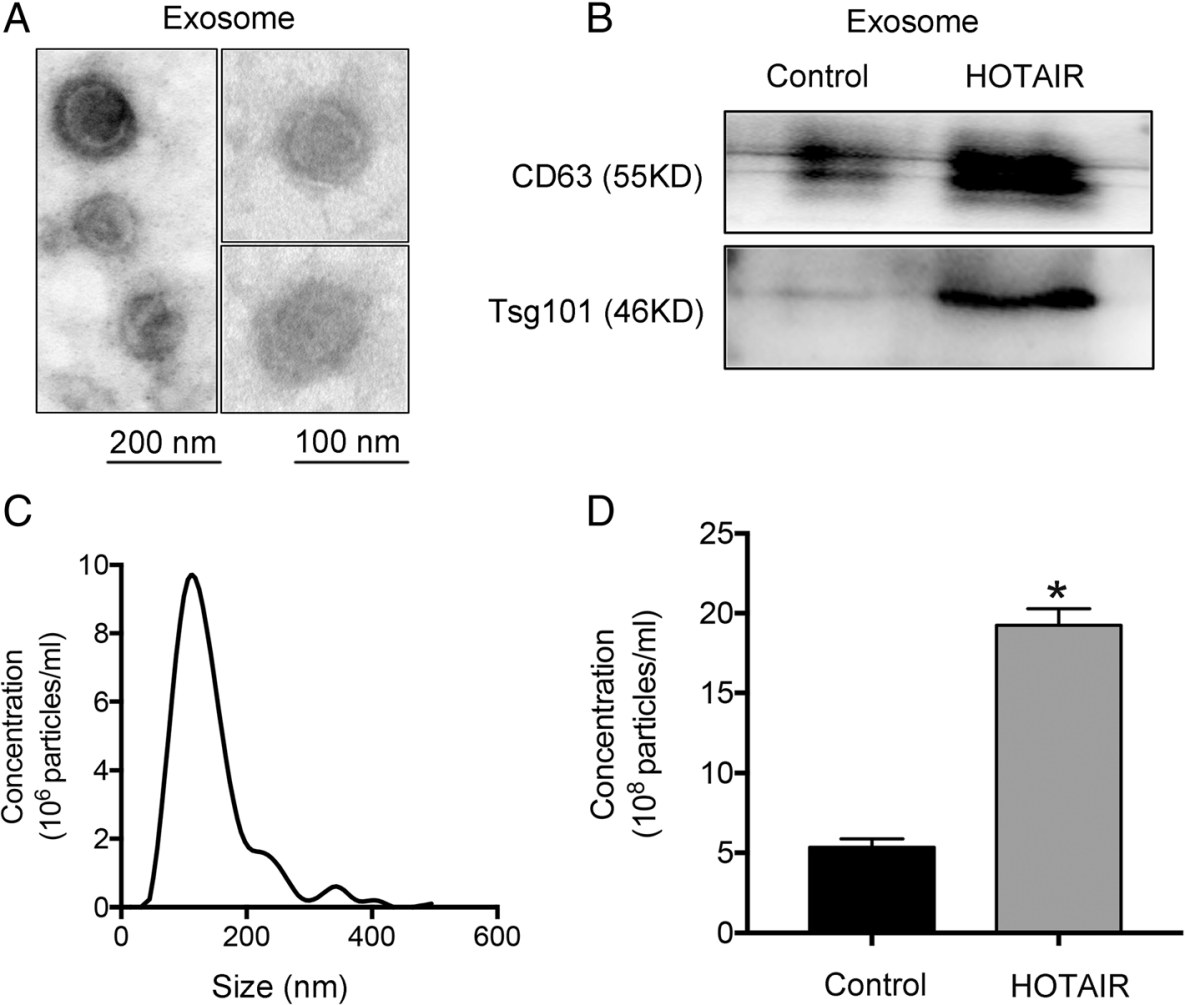
HOTAIR promotes exosome secretion in HepG2 cells. **a–c** Isolated exosomes from HepG2 cells were assessed by **a** transmission electron microscopy, **b** western blotting and **c** NTA. **d** NTA analysis of the effect of HOTAIR on exosome release in HepG2 cells. Data are reported as the mean ± standard error (SD) from three independent experiments, t-test *P-value < 0.05

To detect exosome secretion, we first isolated exosomes from HCC cell culture medium using ultracentrifugation. We then analyzed the purified exosomes using transmission electron microscopy. As shown in Fig. 2a, exosomes are extracellular vesicles with a double membrane, and are 50–100 nm in diameter. To analyze the effect of lncRNA HOTAIR on exosome secretion, we constructed HOTAIR overexpression cell line by transfection pcDNA3.1-HOTAIR in HepG2 cells. The transfection efficiency was about 56-fold compared with negative control (Additional file 1: Figure S1a). Subcellular fractionation and real-time PCR analysis showed that HOTAIR was mainly located in the cytoplasm of HCC cells, which indicated that HOTRAIR may function in cytoplasm in HCC cells (Additional file 1: Figure S1b). Then we detected the exosome markers CD63 and TSG101 in exosomes purified from the culture medium of cells overexpressing HOTAIR compared with that in the negative control. The results showed that overexpression of HOTAIR could increase the secretion of exosomes containing CD63 and TSG101 (Fig. 2b). In addition, NTA indicated that the sizes of released exosomes were about 100 nm (Fig. 2c). We also found that when HepG2 cells overexpressed HOTAIR, they secreted more exosomes (Fig. 2d).These results suggested an important role of HOTAIR in promoting exosome secretion from HCC cells.

### HOTAIR enhances the transport of MVBs towards the plasma membrane

**Fig. 3:**
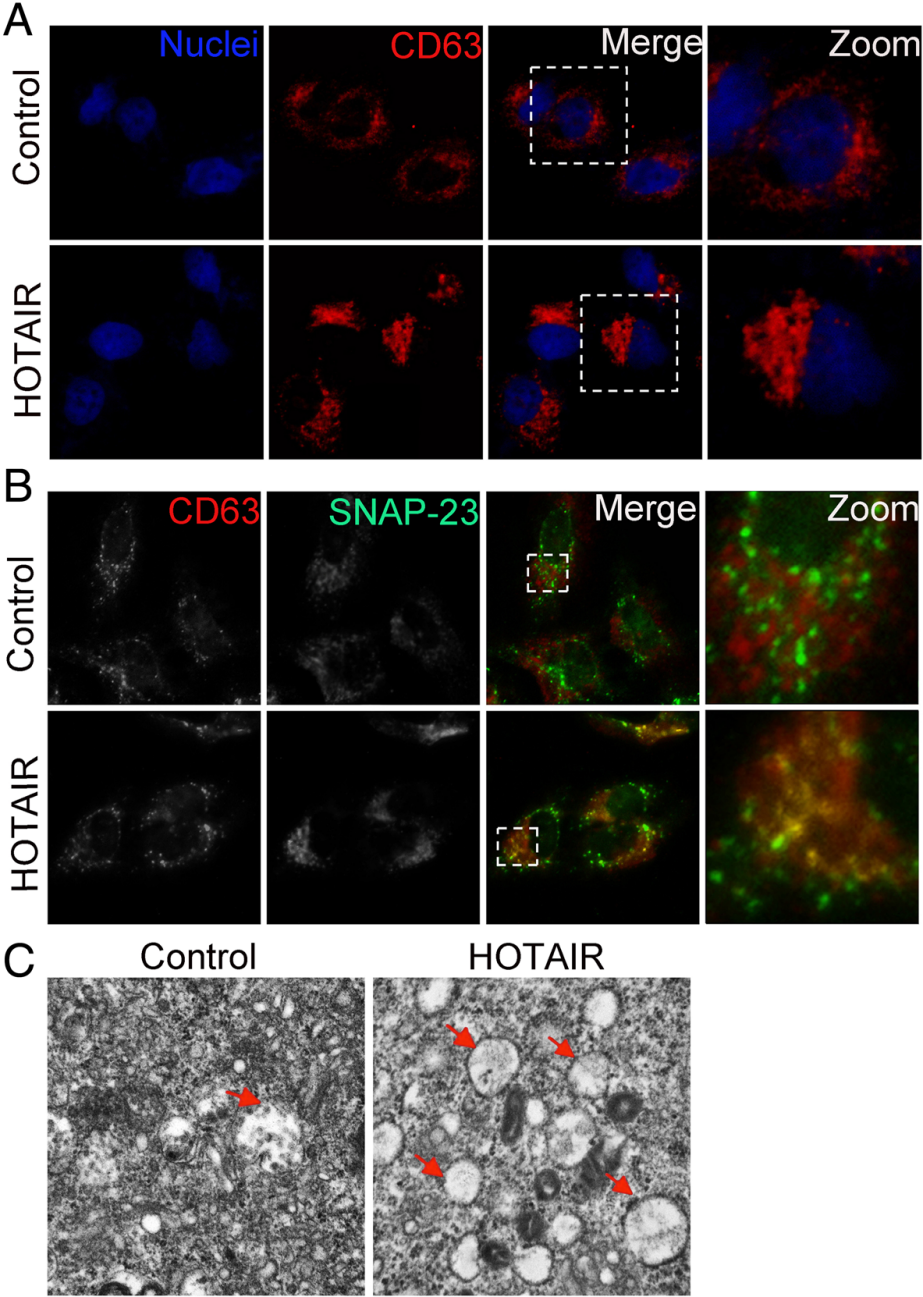
HOTAIR enhances the transport of MVBs towards the plasma membrane. **a** Confocal microscopy analysis of CD63 (red) in HepG2 cells transfected with pcDNA3.1-HOTAIR. Nuclei were stained with DAPI. **b** Confocal co-localization analysis of CD63 (red) and SNAP23 (green) in HepG2 cells transfected with pcDNA3.1-HOTAIR. The rectangular box indicates the small punctate structures where CD63 and SNAP-23 were co-localized. **c** Electron microscopy images showing MVBs in HepG2 cells transfected with pcDNA3.1 or pcDNA3.1-HOTAIR. Red arrows indicated MVBs

To determine the mechanisms by which HOTAIR influences exosome release, we analyzed the process of exosome generation intracellularly. Before exosomes are secreted into the extracellular environment, they are contained within MVBs, which are transported along microtubules to the plasma membrane [25]. Consequently, the effect of HOTAIR on the transport of MVBs was studied. Previously, CD63 has been used as a marker of MVBs. Overexpression of HOTAIR resulted in CD62 being distributed further away from the nucleus (Fig. 3a). In addition, SNAP23 is part of the SNARE complexes that are located mainly in the plasma membrane and regulate MVB docking and fusion with the plasma membrane. The results showed that overexpression of HOTAIR also increased the colocalization of SNAP23 with CD63 (Fig. 3b). Electron microscopy results showed that MVBs were more abundant in HOTAIR overexpressing cells that in the negative control cells (Fig. 3c). Collectively, these results demonstrated that HOTAIR induces the transport of MVBs towards the plasma membrane.

### HOTAIR regulates the expression and the localization of RAB35

**Fig. 4:**
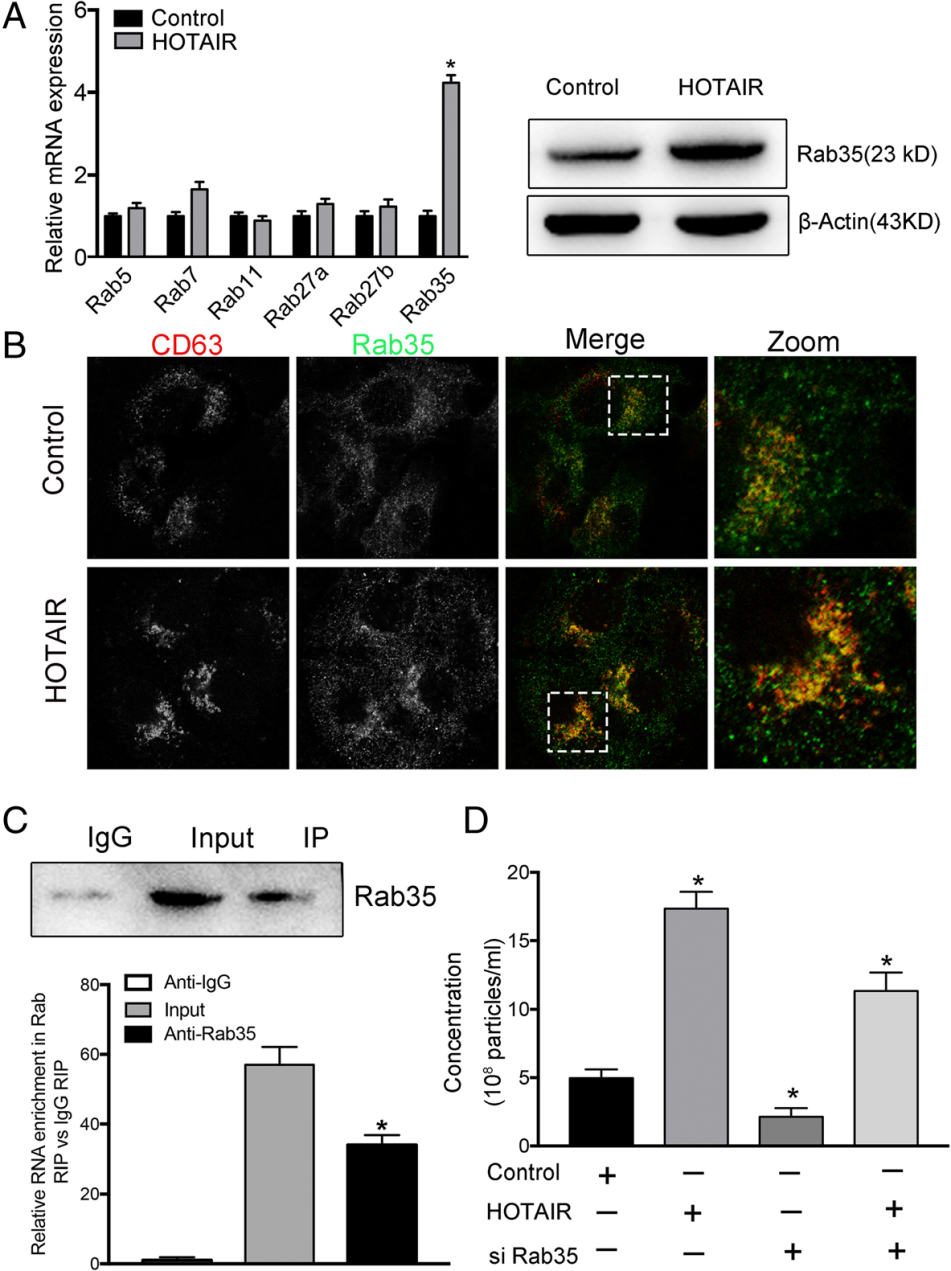
HOTAIR regulates the expression and the localization of RAB35. **a** Real-time PCR analysis of the mRNA expression of RAB5, RAB7, RAB11, RAB27A, RAB27B, and RAB35, which encode GTPases involved in the release of exosomes, in HOTAIR overexpressing HepG2 cells. **b** Western blotting analysis of RAB35 protein levels from the above cells. **c** Confocal co-localization analysis of CD63 (red) and RAB35 (green) in HepG2 cells transfected with pcDNA3.1or pcDNA3.1-HOTAIR. The rectangular box indicates the small punctate structures where CD63 and RAB35 were co-localized. **d** RIP assays were performed using RAB35 antibody or control IgG antibody in HepG2 cells, and then real-time PCR analysis was used to measure the enrichment degrees of HOTAIR coprecipitated RNA. **e** NTA analysis of exosome secretion in HepG2 cells co-transfected with pcDNA3.1-HOTAIR and si-Rab35. Data are reported as the mean ± standard error (SD) from three independent experiments, t-test *P-value < 0.05

To further investigate the molecular mechanisms by which HOTAIR affects the motility of MVBs, we analyzed members of the Rab GTPase family. Previous studies have found that several Rab GTPases are located in a subcellular position coincident with MVBs and mediate MVB transport along microtubules [14, 15]. Our previous review summarized the Rab GTPases involved in the release of exosomes, which include RAB5, RAB7, RAB11, RAB27a, RAB27b, and RAB35 [5]. We screened the expression of these Rab GTPases using real-time PCR. The results showed that RAB35 was the most upregulated Rab GTPase gene in response to HOTAIR overexpression in HCC cells (Fig. 4a). Consistent with this result, overexpression of HOTAIR increased the abundance of the of RAB35 protein, as assessed using western blotting analysis (Fig. 4b). The same effect was demonstrated in Huh7 cells (Additional file 2: Figure S2a-b). Next, we investigated whether HOTAIR influenced the subcellular localization of RAB35. Our results indicated that overexpression of HOTAIR could induced stronger staining of RAB35 and an increased the colocalization of RAB35 with CD63, which suggested that HOTAIR regulated the localization of RAB35 at MVBs and upregulated RAB35 expression (Fig. 4c). RIP assay results showed that HOTAIR was significantly enriched by Rab35 antibody compared with control IgG (Fig. 4d). The specific association between HOTAIR and Rab35 was further validated by pull-down assay using in vitro transcribed biotin-labeled HOTAIR (Additional file 2: Figure S2d). Our findings demonstrate a direct interaction between HOTAIR and Rab35. Thus, the main mechanism of HOTAIR is to promote MVBs transport to the plasma membrane. Furthermore, we demonstrated that HOTAIR’s promotion of exosome secretion was eliminated by cotransfection with HOTAIR and RAB35-specific siRNAs (Fig. 4e). The knock-down efficiency of si-RAB35 is detected by western blot (Additional file 2: Figure S2c). By cotransfection assay, we aimed to demonstrated HOTAIR influence exosome secretion via regulation Rab35. These results support the view that HOTAIR promotes the motility of MVBs by regulating the expression and localization of RAB35.

### HOTAIR induces the translocation of VAMP3 and SNAP23

**Fig. 5:**
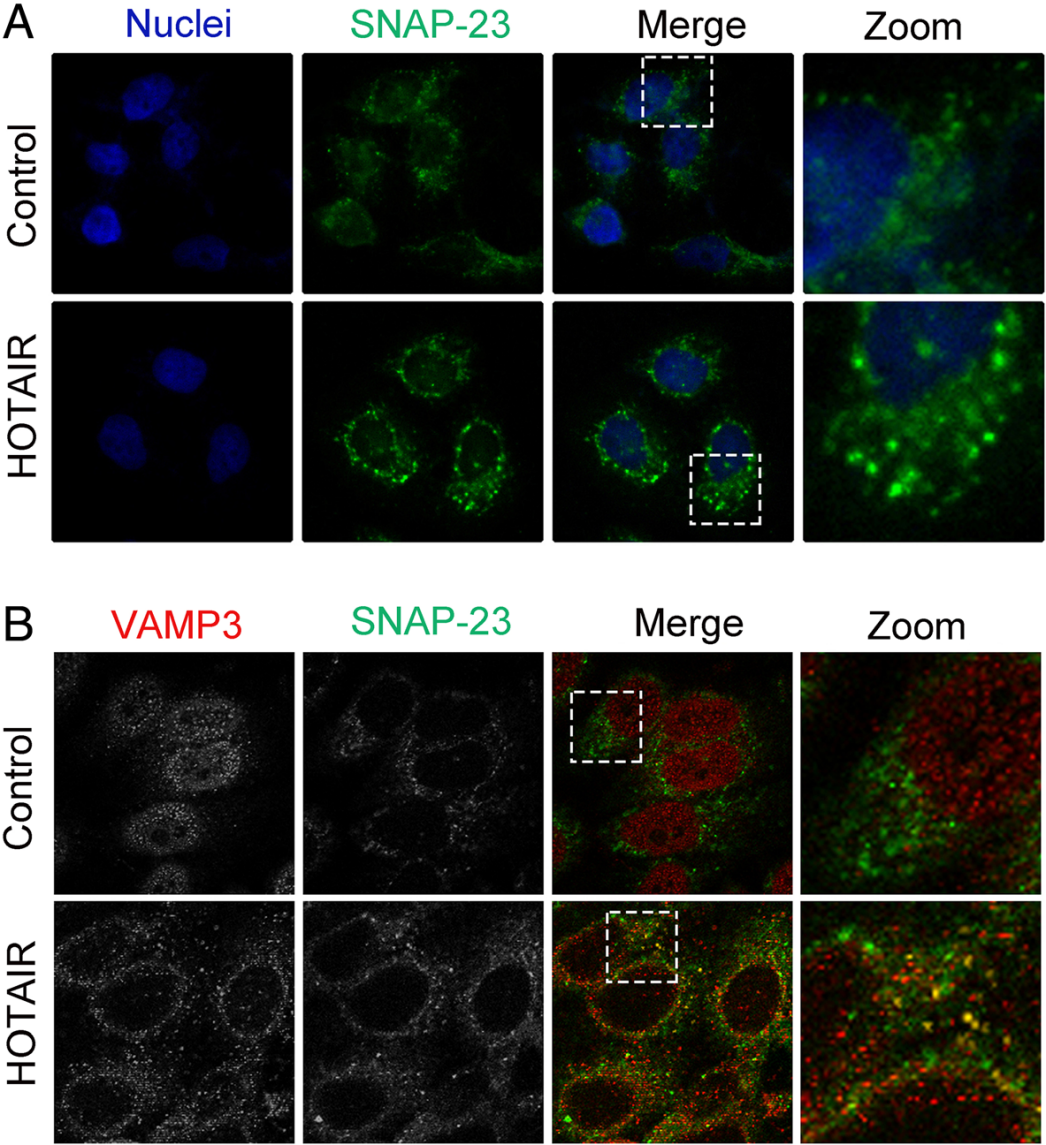
HOTAIR induces the translocation of VAMP3 and SNAP23. **a** Confocal microscopy analysis of SNAP23 (green) in HepG2 cells transfected with pcDNA3.1-HOTAIR. Nuclei were stained with DAPI. **b** Confocal co-localization analysis of VAMP3 (red) and SNAP23 (green) in HepG2 cells transfected with pcDNA3.1-HOTAIR. The rectangular box indicates the small punctate structures where VAMP3 and SNAP23 were co-localized

When MVBs are transported to the cell membrane, they must fuse with the plasma membrane to release the ILVs as exosomes. This process is mediated by SNARE transmembrane proteins. One SNARE molecule on the MVB membrane (v-SNARE) binds to SNAREs on plasma membrane (t-SNARE), forming a stable ternary complex (trans-SNARE) that mediates MVB fusion with the plasma membrane [16]. A previous study indicated that SNAP23, as an important t-SNARE located at plasma membrane, plays a vital role in regulating secretion by binding to VAMP3 as a v-SNARE [19]. Overexpression of HOTAIR induced SNAP23 to become diffusely located at the plasma membrane compared with the negative control (Fig. 5a). Furthermore, overexpression of HOTAIR also promoted an increased colocalization of VAMP3 with SNAP23, which suggested that HOTAIR could induce SNARE complex formation to influence the fusion process of MVBs (Fig. 5b).

### HOTAIR promotes the release of exosome via phosphorylation of SNAP23

**Fig. 6:**
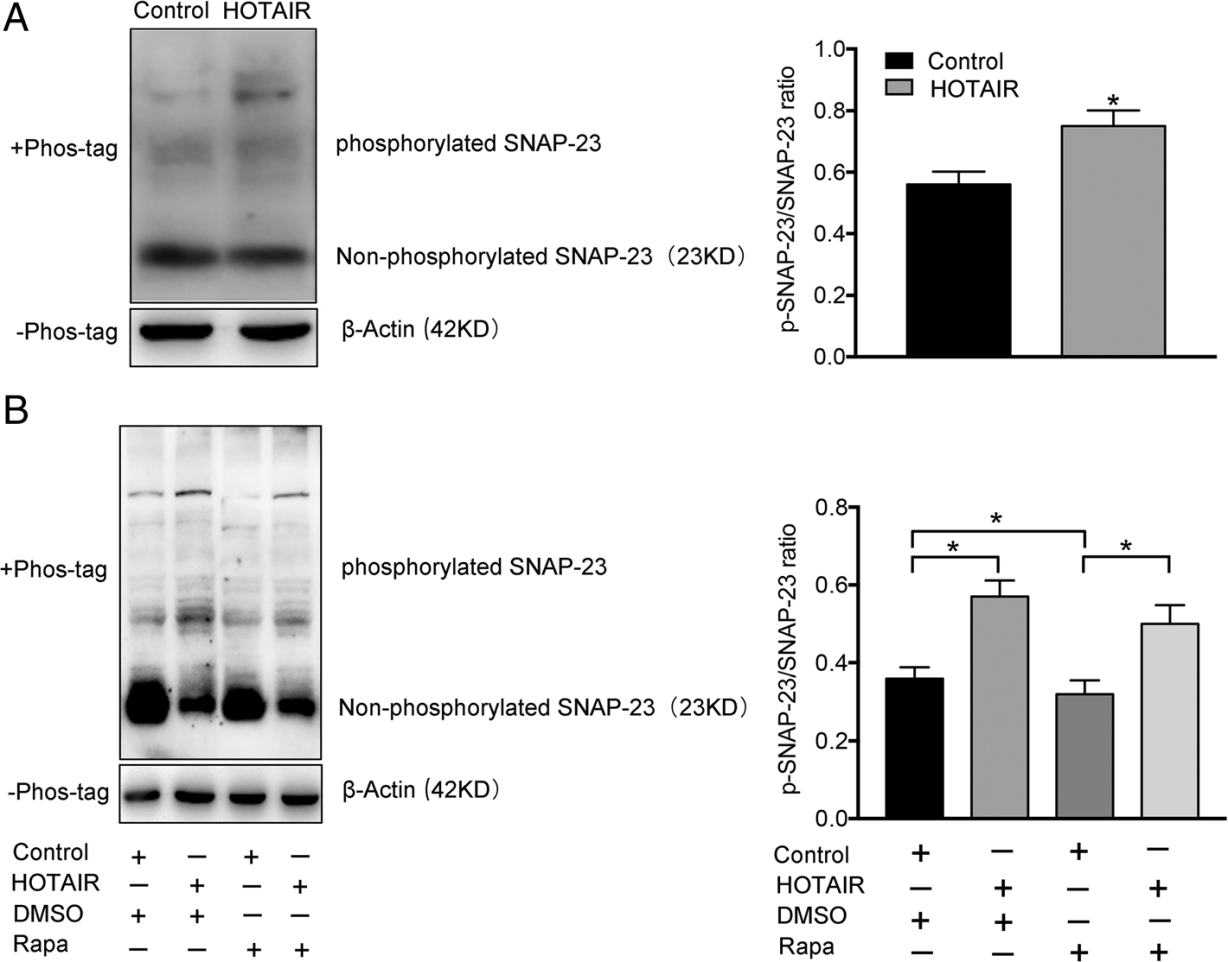
HOTAIR induces phosphorylation of SNAP23 via mTOR signaling. The levels of non-phosphorylated SNAP23 in HepG2 cells as assessed using Phos-tag SDS-PAGE analysis. **a** HepG2 cells were transfected with pcDNA3.1or pcDNA3.1-HOTAIR. **b** HepG2 cells transfected with pcDNA3.1 or pcDNA3.1-HOTAIR were treated with rapamycin (Rapa, 50 nmol/L) or control vehicle (DMSO), respectively. Data are reported as the mean ± standard error (SD) from three independent experiments, t-test *P-value < 0.05

Based on the result that HOTAIR regulates the location of SNAP23, we next tested whether HOTAIR could influence the activity of SNAP23. A previous study indicated that SNAP23 phosphorylation is required for exosome secretion [26]. To test whether HOTAIR could regulate SNPA23 phosphorylation, we performed a phosphorylation assay to detect the level of phosphorylated SNAP23 in HepG2 cells transfected with pcDNA3.1-HOTAIR. As shown in Fig. 6a, overexpression of HOTAIR significantly increased the ratio of p-SNAP23/SNAP23 compared with that in the negative control (Fig. 6a). This result suggested that HOTAIR could induce SNAP23 phosphorylation. We next sought to determine how HOTAIR phosphorylates SNAP23. mTOR signaling is involved in regulating SNARE complexes [27]. Our results showed that overexpression of HOTAIR increased the amount of phosphorylated (p)-mTOR (Additional file 3: Figure S3a). Using rapamycin (an inhibitor of mTOR) to perform a rescue experiment, we found that the effect of HOTAIR in inducing SNPA23 phosphorylation was attenuated when the transfected cells were co-treated with rapamycin (Fig. 6b). Moreover, exosome release induced by overexpression HOTAIR was significantly decreased after rapamycin-induced inhibition of mTOR (Additional file 3: Figure S3b). These results suggested that HOTAIR could promote the release of exosomes via phosphorylation of SNAP23.

## Discussion

Cells can secrete many extracellular vesicles, such as microvesicles (shedding vesicles) and exosomes. The biogenesis mechanisms of these extracellular vesicles are different. Microvesicles originate from evagination of the plasma membrane, while exosomes originate from the endosomal system as ILVs and are released via the fusion of MVBs with the cell membrane [11]. Alternatively, MVBs can fuse with lysosomes to degrade their content. Although the detailed mechanism of how MVBs finally fuse with the lysosome or plasma membrane remains unclear, there is some evidences that suggested that the final fate of MVBs can be influenced by certain special conditions [28]. In tumor progression, tumor cells face the loss of cellular homeostasis, such as via hypoxia, starvation, inflammation, and metabolic stress. Hypoxia is a common feature of solid tumors. Interestingly, previous studies found that hypoxia could not only promote the release of exosomes [8, 29, 30], but also altered the contents of tumor-derived exosomes, which affected tumor progression through communication between the tumor cells and their microenvironment [29]. Moreover, aerobic glycolysis, also termed the Warburg effect, is the characteristic of glucose metabolism in tumor cells in response to metabolic stress. Wei et al. found that pyruvate kinase M1/2 (PKM2), the key enzyme of aerobic glycolysis, plays a vital role in promoting the release of exosomes from tumor cells [18]. Although the phenomenon of increasing exosome secretion is observed during tumorigenesis, the molecular mechanisms controlling tumor exosome release remain unclear.

Recently, lncRNAs have become a focus in the field of cancer research. Many studies have demonstrated that lncRNAs are involved in the biological functions of tumor cells, such as proliferation, invasion, metastasis, immunological function, metabolism, and drug resistance [31, 32]. However, few studies have explored the relationship between lncRNAs and the regulation of exosome secretion from tumor cells. In the present study, we analyzed the potential role of lncRNA HOTAIR in the exocytosis of exosomes. First, our GSEA analysis demonstrated an enrichment of exosome secretion-related genes in the group of patients showing relatively high HOTAIR expression. Second, using Nanosight analysis, we demonstrated that overexpression of HOTAIR promoted exosome secretion from HCC cells. Third, based on the intracellular process of exosome secretion, we showed that HOTAIR facilitated the transport of MVBs to the plasma membrane. Collectively, these results demonstrated that lncRNA HOTAIR promotes exosome secretion and the motility of MVBs in HCC cells, which suggest a positive link between HOTAIR and exosome release.

Exosome secretion is a multi-step process regulated by a series of molecules and involves the transport of MVBs, and their docking and fusion with the plasma membrane. Given that HOTAIR could promote the transport of MVBs in HCC cells, we aimed to explore the molecular mechanism underlying this complex process. Previous studies showed that several Rab GTPases, such as RAB5, RAB7, RAB11, RAB27a, RAB27b, and RAB35, play a vital role in regulating MVB transport and influence the docking process. Our results showed that overexpression of HOTAIR significantly increased RAB35 expression, suggesting that this is the Rab GTPase that regulates the transport of MVBs in association with HOTAIR. Moreover, overexpression of HOTAIR induced RAB35 to locate at the MVB membrane. In 2010, Hsu et al. provided a basis for understanding RAB35’s function in the central nervous system. RAB35 localizes to the surface of oligodendroglia in a GTP-dependent manner, leading to the intracellular release of endosomal vesicles and reduced exosome secretion [14]. Another study showed that RAB35’s function in Hela cells was consistent with that in oligodendroglia cells, which implied that RAB35 is an important regulator in the control MVB transport [33]. Our findings suggested that HOTAIR induces the transport of MVBs by regulating RAB35 expression and its subcellular localization.

MVBs fusion with the plasma membrane is the final step of exosome secretion. The SNARE complex mediates this fusion process, contributing to release ILVs as exosomes. During fusion, one v-SNARE on the MVB membrane binds to the t-SNAREs on the plasma membrane, forming a SNARE complex, which controls the release of exosomes [16]. SNAP23 is a t-SNARE molecule that has an important function in mediating exosome secretion. In vascular endothelial cells, Zhu et al. found that atherogenic oscillatory shear (OS) could both promote v-SNARE VAMP3 and t-SNARE SNAP23 expression, and modulate the subcellular localization of VAMP3 and SNAP23, which contributed to microRNA-mediated endothelial cell-smooth muscle cell communication [34]. Our study showed that HOTAIR induced the diffuse location of SNAP23 at the plasma membrane. In addition, we found that HOTAIR overexpression promoted the colocalization of VAMP3 with SNAP23. These results suggested that HOTAIR participates in the formation of the SNARE complex to promote MVB fusion with the plasma membrane. Previous studies demonstrated that phosphorylation of SNAP23 is required for exocytosis and promotes its association with other SNARE proteins [35]. However, it was unknown whether lncRNA HOTAIR could affect the phosphorylation of SNAP23 in HCC cells. Our results showed that HOTAIR could induce the phosphorylation of SNAP23 via activation of mTOR. These results indicated the complex mechanisms by which HOTAIR regulates SNARE complexes to promote exosome secretion.

The final fate of MVBs that are not transported to the plasma membrane to release exosomes appears to be fusion with lysosomes or autophagosomes to degrade their content. The mechanism controlling the balance between degradation and secretion for MVBs remains poorly understood. However, further exploration of this balance will lead to a deeper understanding of the characteristics of tumor cells. Several studies have found that the fate of MVBs can be modulated under specific stimuli. For example, Wei et al. revealed that glycolysis promotes the release of exosomes from tumor cells via phosphorylation of SNAP23 [18]. In addition, ISGylation (the covalent addition of interferon-stimulated gene 15 (ISG15)) is a ubiquitin-like modification that could induce MVB protein aggregation and degradation to impair exosome secretion [36]. Evidence has emerged that the balance between exosome secretion and autophagy, which as a conserved catabolic process, supports tumor cell survival under stress [37]. The fusion of MVBs with the autophagosome would promote MVB degradation and inhibit exosome secretion [11, 37]. Interestingly, this balance seems to be paradoxical in tumors. Both autophagy and exosome secretion become active during tumor progression, such as under hypoxia stress [30, 38]. We hypothesized that the molecular mechanisms of this tumor cell-specific balance are complex and thus require further exploration.

## Conclusion

**Fig. 7:**
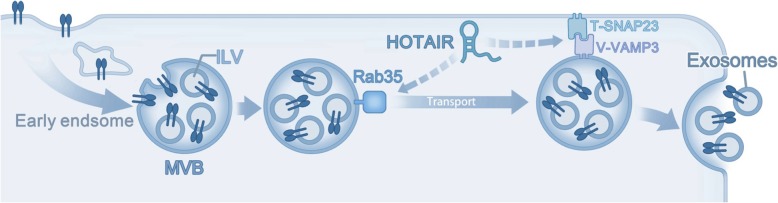
Proposed model for the role of HOTAIR in the regulation of exosome secretion. HOTAIR accelerates MVB transport by inducing RAB35 expression and localization to MVBs. In addition, HOTAIR promotes MVB fusion by regulating the location and phosphorylation of SNAP23 to form the SNARE complex

In summary, our findings demonstrated a vital role of lncRNA HOTAIR in regulating exosome secretion from HCC cells (Fig. 7). HOTAIR promoted the release of exosomes by inducing the transport of MVBs to the plasma membrane. HOTAIR regulates RAB35 expression and localization, which controls the docking process. Moreover, HOTAIR facilitated the final step of fusion by affecting the colocalization and activity of VAMP3 and SNAP23. Our study highlights a novel function of lncRNA HOTAIR in regulating exosome secretion and provides an insight into the participation of lncRNAs in exosome-mediated communication in HCC.

## Additional files

Additional file 1: **Figure S1. a** Real-time PCR analyze the transfection efficiency of HOTAIR overexpression in HepG2 cells. **b** Nuclear and cytoplasmic RNA levels of HOTAIR was measured by real-time PCR after subcellular fractionation in HepG2 cells, t-test *P-value < 0.05. (JPG 606 kb)

Additional file 2: **Figure S2. a** Real-time PCR analysis of the mRNA expression of RAB5, RAB7, RAB11, RAB27A, RAB27B, and RAB35, which encode GTPases involved in the release of exosomes, in HOTAIR overexpressing Huh7 cells. **b** Western blotting analysis of RAB35 protein levels from the above cells. **c** Western blot were used to examine knockdown efficiency of Rab35 in HepG2 cells transfected with Rab35 special siRNAs. **d** Pull-down assay showed that biotin-labeled HOTAIR associates with recombinant Rab35, which antisense HOTAIR was used as the negative control RNA in pull-down assay, t-test *P-value < 0.05. (JPG 1373 kb)

Additional file 3: **Figure S3.** mTOR mediates HOTAIR’s promotion of the release of exosomes via phosphorylation of SNAP23. **a** Western blotting analysis of mTOR and p-mTOR in HepG2 cells transfected with pcDNA3.1 or pcDNA3.1-HOTAIR and treated with Rapa or DMSO, respectively. **b** NTA analysis of exosome secretion from the above culture medium of HepG2 cells. Data are reported as the mean ± standard error (SD) from three independent experiments, t-test *P-value < 0.05. (JPG 974 kb)

## Abbreviations

ESCRT: Endosomal sorting complex required for transport
GSEA: Gene set enrichment analysis
HCC: Hepatocellular carcinoma
HOTAIR: HOX transcript antisense RNA
ILVs: Intraluminal vesicles
LncRNAs: Long non-coding RNAs
MVBs: Multivesicular bodies
NTA: Nanoparticle tracking analysis
SNAP: Synaptosomal-associated protein
SNARE: Soluble N-ethylmaleimide-sensitive fusion factor attachment protein receptor
VAMP: Vesicle-associated membrane protein
