# Supplementary figures and images for: Long non-coding RNA HOTAIR promotes exosome secretion by regulating RAB35 and SNAP23 in hepatocellular carcinoma
Source: Mol Cancer. 2019 Apr 3;18:78. doi: 10.1186/s12943-019-0990-6 (PMC6446409; doi:10.1186/s12943-019-0990-6)

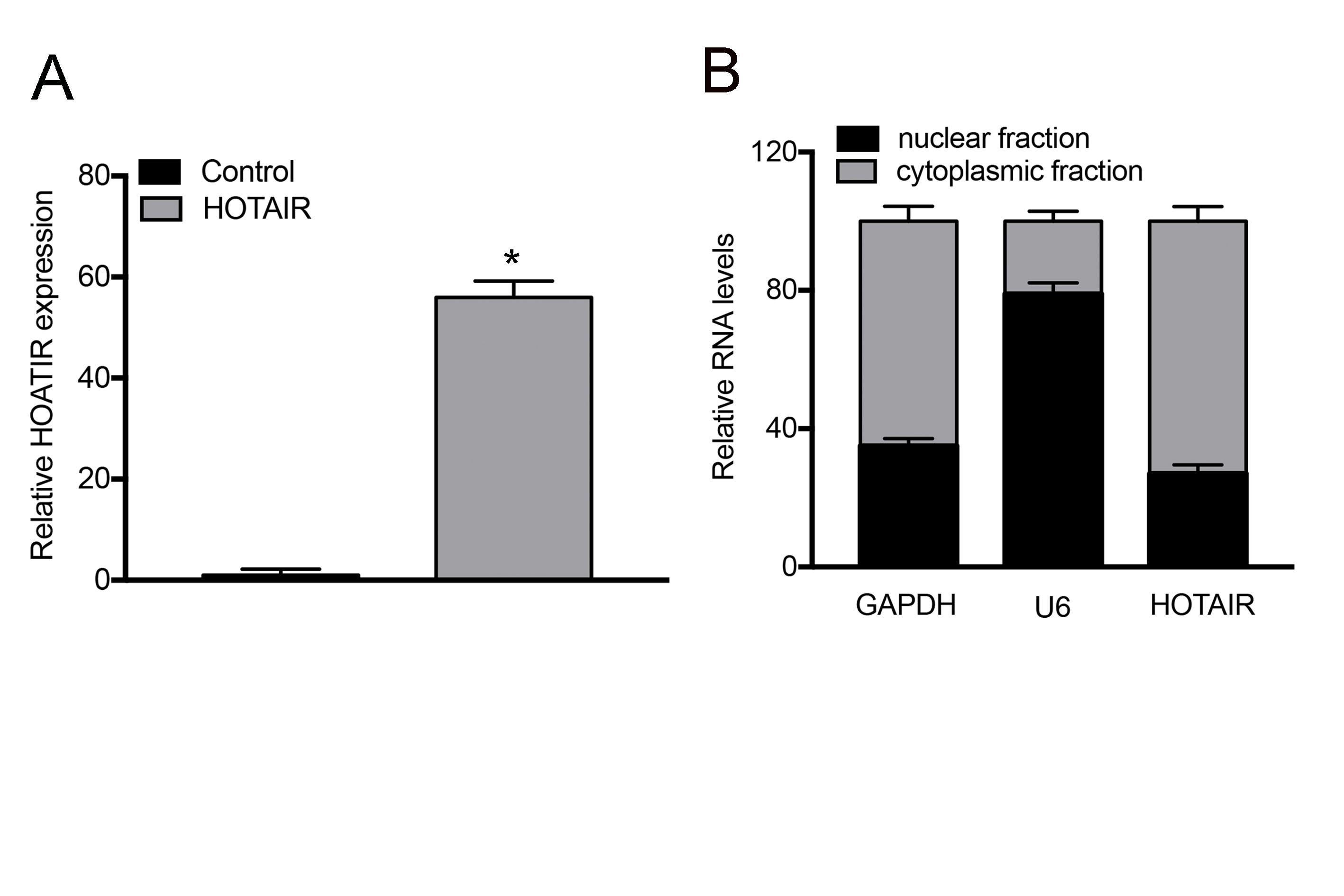

Supplement: Supplementary file 1 — Figure S1. a Real-time PCR analyze the transfection efficiency of HOTAIR overexpression in HepG2 cells. b Nuclear and cytoplasmic RNA levels of HOTAIR was measured by real-time PCR after subcellular fractionation in HepG2 cells, t-test *P-value < 0.05. (JPG 606 kb) [file 12943_2019_990_MOESM1_ESM.jpg]

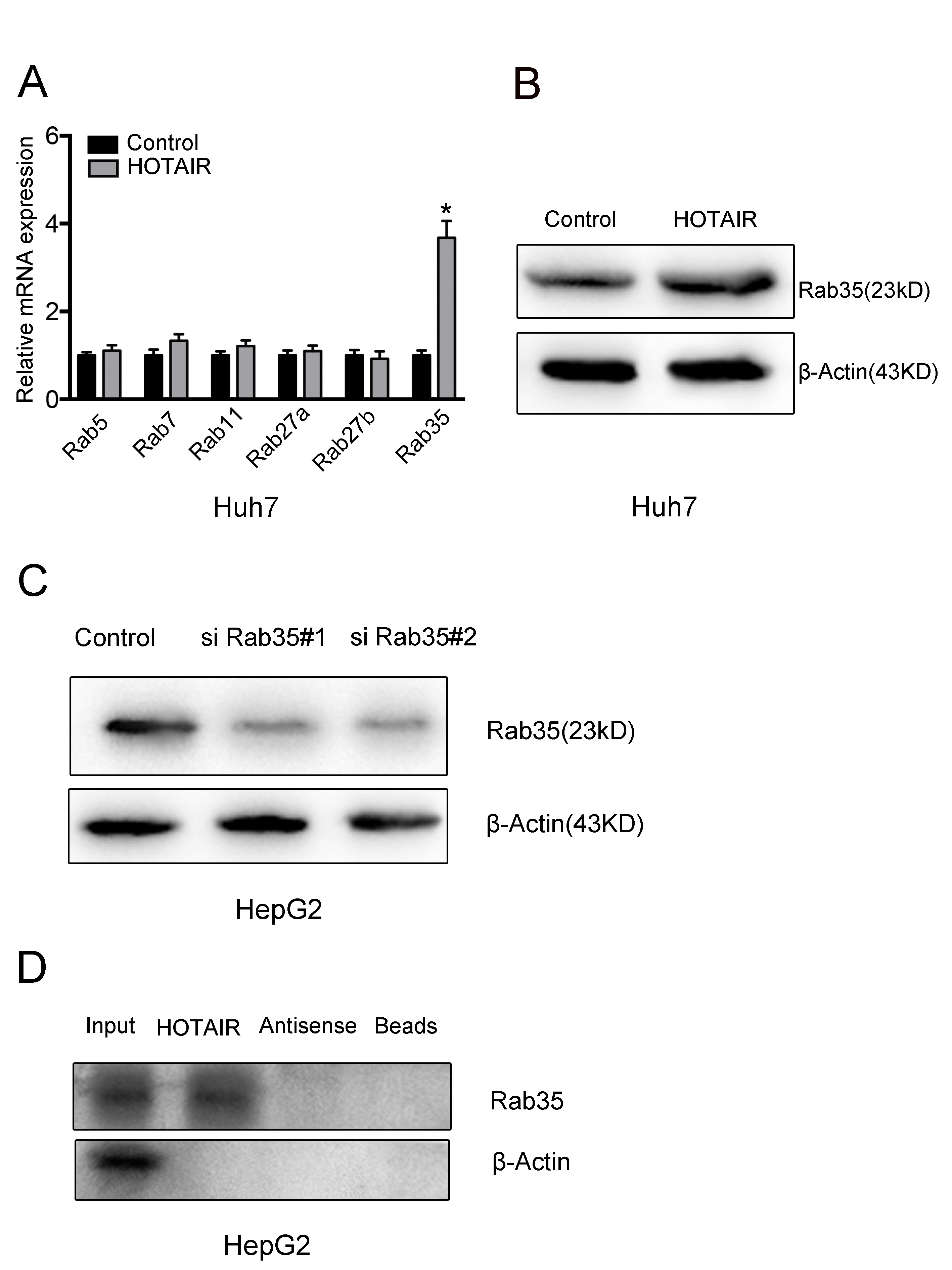

Supplement: Supplementary file 2 — Figure S2. a Real-time PCR analysis of the mRNA expression of RAB5, RAB7, RAB11, RAB27A, RAB27B, and RAB35, which encode GTPases involved in the release of exosomes, in HOTAIR overexpressing Huh7 cells. b Western blotting analysis of RAB35 protein levels from the above cells. c Western blot were used to examine knockdown efficiency of Rab35 in HepG2 cells transfected with Rab35 special siRNAs. d Pull-down assay showed that biotin-labeled HOTAIR associates with recombinant Rab35, which antisense HOTAIR was used as the negative control RNA in pull-down assay, t-test *P-value < 0.05. (JPG 1373 kb) [file 12943_2019_990_MOESM2_ESM.jpg]

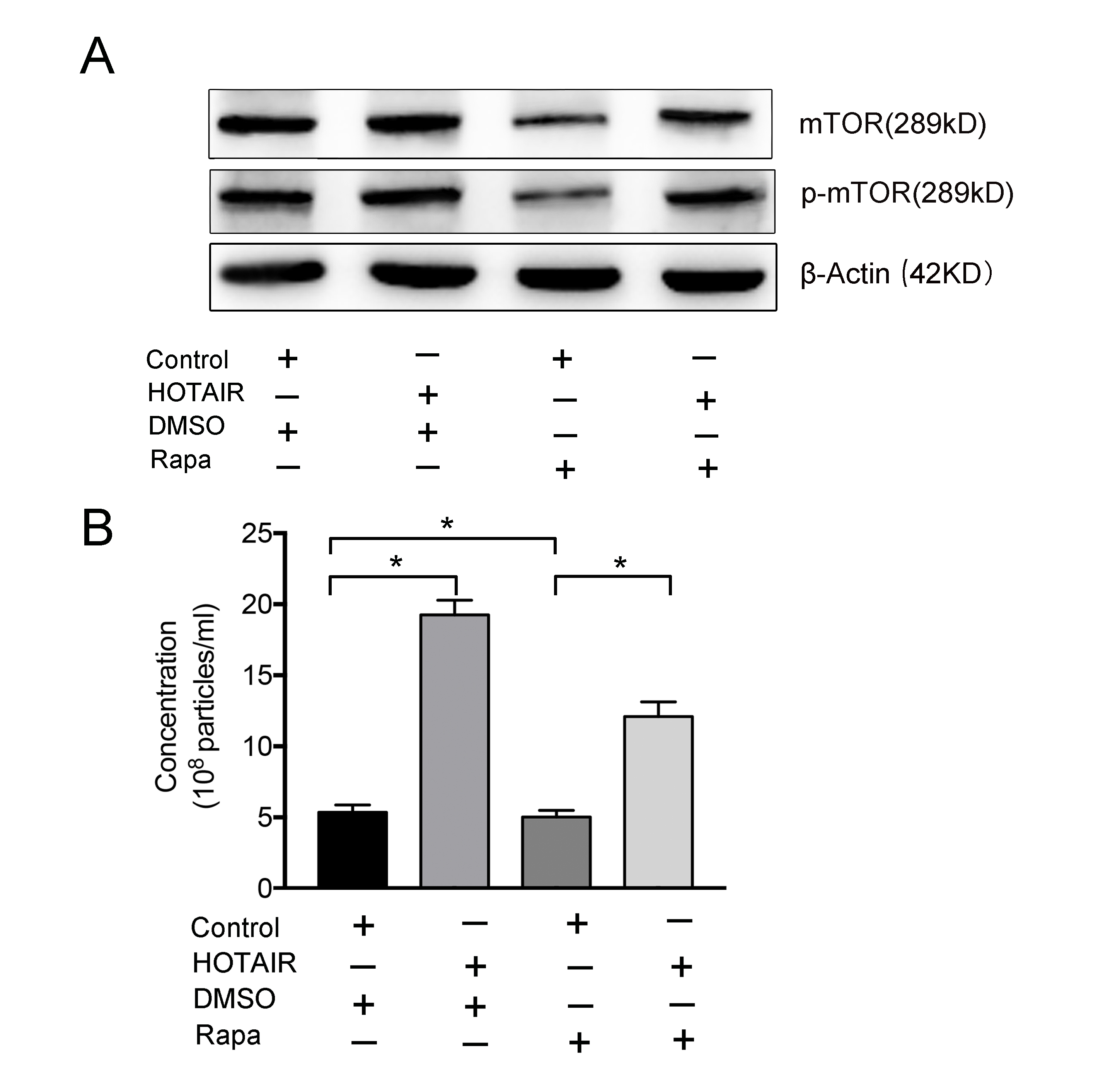

Supplement: Supplementary file 3 — Figure S3. mTOR mediates HOTAIR’s promotion of the release of exosomes via phosphorylation of SNAP23. a Western blotting analysis of mTOR and p-mTOR in HepG2 cells transfected with pcDNA3.1 or pcDNA3.1-HOTAIR and treated with Rapa or DMSO, respectively. b NTA analysis of exosome secretion from the above culture medium of HepG2 cells. Data are reported as the mean ± standard error (SD) from three independent experiments, t-test *P-value < 0.05. (JPG 974 kb) [file 12943_2019_990_MOESM3_ESM.jpg]
